# Principles of Immunotherapy: Implications for Treatment Strategies in Cancer and Infectious Diseases

**DOI:** 10.3389/fmicb.2018.03158

**Published:** 2018-12-21

**Authors:** Krupa Naran, Trishana Nundalall, Shivan Chetty, Stefan Barth

**Affiliations:** ^1^Medical Biotechnology and Immunotherapy Unit, Institute of Infectious Disease and Molecular Medicine, Faculty of Health Sciences, University of Cape Town, Cape Town, South Africa; ^2^South African Research Chair in Cancer Biotechnology, Department of Integrative Biomedical Sciences, Faculty of Health Sciences, University of Cape Town, Cape Town, South Africa

**Keywords:** immunotherapy, T cell therapy, antibody therapy, cancer, infectious diseases

## Abstract

The advances in cancer biology and pathogenesis during the past two decades, have resulted in immunotherapeutic strategies that have revolutionized the treatment of malignancies, from relatively non-selective toxic agents to specific, mechanism-based therapies. Despite extensive global efforts, infectious diseases remain a leading cause of morbidity and mortality worldwide, necessitating novel, innovative therapeutics that address the current challenges of increasing antimicrobial resistance. Similar to cancer pathogenesis, infectious pathogens successfully fashion a hospitable environment within the host and modulate host metabolic functions to support their nutritional requirements, while suppressing host defenses by altering regulatory mechanisms. These parallels, and the advances made in targeted therapy in cancer, may inform the rational development of therapeutic interventions for infectious diseases. Although “immunotherapy” is habitually associated with the treatment of cancer, this review accentuates the evolving role of key targeted immune interventions that are approved, as well as those in development, for various cancers and infectious diseases. The general features of adoptive therapies, those that enhance T cell effector function, and ligand-based therapies, that neutralize or eliminate diseased cells, are discussed in the context of specific diseases that, to date, lack appropriate remedial treatment; cancer, HIV, TB, and drug-resistant bacterial and fungal infections. The remarkable diversity and versatility that distinguishes immunotherapy is emphasized, consequently establishing this approach within the armory of curative therapeutics, applicable across the disease spectrum.

## Introduction

Numerous host factors which constitute the immune system influence treatment outcomes and are accountable for disease progression or regression. Immunotherapy is collectively defined as a therapeutic approach that targets or manipulates the immune system (Papaioannou et al., 2016). Ultimately, immunotherapy aims to harness the host’s adaptive and innate immune response to effectuate long-lived elimination of diseased cells and can be categorized broadly into passive (including adoptive and antibody-based) and active (including vaccine therapy and allergen-specific) approaches. Passive-mediated immunotherapy involves the administration of *ex vivo*-generated immune elements (antibodies, immune cells) to patients and does not stimulate the host immune response, while active immunotherapy induces the patient’s immune response and results in the development of specific immune effectors (antibodies and T cells) (Tur and Barth, 2014).

Consequently, these targeted therapies advance host cellular responses to disease by stimulating immune responses, targeting disease-causing virulence factors and improving immunological memory (Zumla et al., 2016b). As such, several types of targeted therapies have been approved for cancer treatment and act by blocking regulatory biochemical pathways or mutant proteins, essential to tumor growth and maintenance and have reduced toxic side effects as they act with precision on diseased cells with little collateral tissue damage (Wykes and Lewin, 2018). As such, “immunotherapy” is almost exclusively, and somewhat biasedly, associated with the treatment of cancer. In this review, clinically relevant examples of adoptive therapies are provided to emphasize the versatility of their corresponding general principles of action for both non-communicable and infectious diseases.

## T Cell Therapies

**FIGURE 1:**
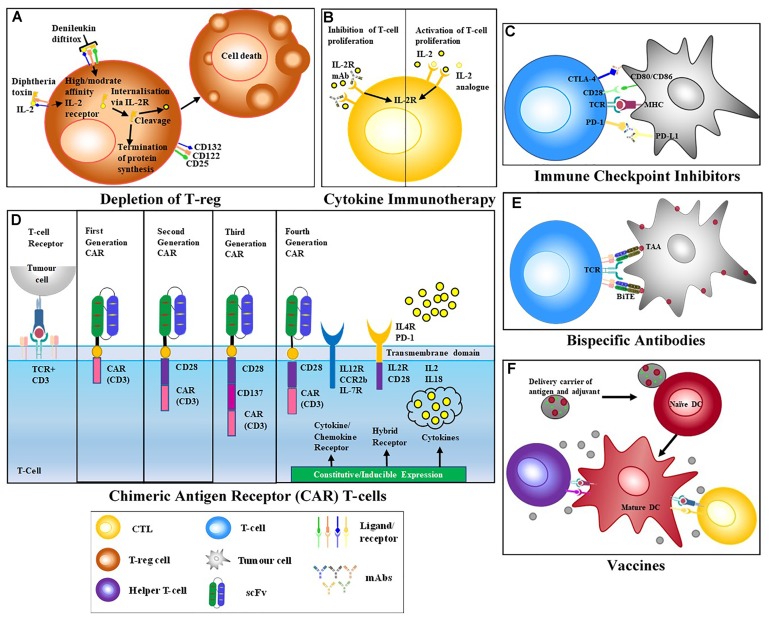
T cell-activating Therapeutic Strategies. **(A)** Treg depletion – biologics such Denileukin diftitox (DD) bind to target receptors on suppressor cells and initiate apoptosis via down-stream signaling. **(B)** Cytokine therapy – addition of pro-inflammatory cytokines increases immune activation while the addition of anti-inflammatory cytokines reduces immune activation. MAbs specific for cytokine receptors may also be used to block cytokine stimulation of the immune system. **(C)** Immune checkpoint blockade – mAbs block the interaction of inhibitory receptors CTLA-4 and PD-1, resulting in the activation of effector T cells (QYResearch) **(D)** Chimeric antigen receptors (CARs) T cells are modified T cells with a recombinant receptor; usually a scFv that redirects the specificity of effector T cells. First generation CARs that only comprised an activation domain were prone to anergy. Due to this signaling failure, second and third generation CARs, incorporating a CD3 chain and cytoplasmic domain of a co-stimulatory receptor, like CD28 were generated. Fourth generation CARs also included constitutive or inducible expression of co-receptors or soluble cytokines together with T cell activating CAR (Golubovskaya and Wu, 2016). **(E)** Bispecific antibodies containing two binding arms one specific for a target antigen and a second arm specific for CD3, thereby bringing T cells into close proximity to target cells and activating T cells while bypassing the need for MHC restricted engagement. **(F)** Vaccines – Introduction of non-infectious component to stimulate activation of T cells and development of memory immune cells.

T cells initiate potent and specific immune responses against foreign antigens. Strategies that activate these immune responses, broadly define T cell therapies and are often developed in combination with monoclonal antibodies (mAbs) (Figure 1).

### Vaccines

Vaccination represents the first form of host-directed immunotherapy to be introduced and includes various categories (Table 1). Most vaccines work by introducing a non-infectious version of a disease-causing microbe into an individual, thereby providing a better stimulus for the activation of disease-specific T cells and the development of immunological memory. Memory immune cells are able to rapidly kill microbes and prevent infection. While this type targeted therapy has been able to eradicate smallpox and drastically reduce the disease burden of multiple infectious agents such as rabies, typhoid, cholera, hepatitis and more, it has been far less effective against cancer and chronic infectious diseases such as human immunodeficiency virus (HIV). The combination of therapeutic vaccines and other immune-based therapies may provide improved efficacy (Waldmann, 2003).

**Table 1:** Vaccine types and examples.

Type	Description	Advantages	Disadvantages	Disease	Reference
Live attenuated	Less pathogenic strain of microbe	Induction of long lived responses	Adverse effects in immune-compromised	MMR, Smallpox	World Health Organization, 2017
Inactivated	Pathogens killed through chemical treatment or heat	Cannot replicate	Often induces weaker immune responses than other methods	Cholera	Bi et al., 2017
Subunit	A vaccine designed to induce immune responses to the most dominant epitopes of a pathogen	High level of safety	Multiple doses are usually required	Hepatitis B	Van Den Ende et al., 2017
Toxoid	Induces an immune response to the pathogens toxin	Strong antibody response and long-lasting antigen specific memory	Booster doses are often required	Diphtheria	World Health Organization, 2017
Conjugate	A strong antigen (often a protein) covalently attached to a weak antigen (often a bacterial polysaccharide)	Safe for use in infants. Long lasting immune responses	Expensive to produce	Bacterial Meningitis	Wasserman et al., 2018
DNA	Fragments of DNA encoding antigens for specific pathogens are injected for endogenous production	Non-infectious, no cold chain required	Limited to protein antigen production	Experimental	Vetter et al., 2018
Recombinant	Recombinant DNA delivered through bacterial or viral vaccine vectors	Strong immune responses	Anti-vector immunity can lead to adverse effects	HPV	Sipp et al., 2018


Following the discovery of Edward Jenner’s smallpox vaccine in 1796 (Nabel, 2013) vaccination, especially against viral infections, has been the leading practice of infection prevention. Developed in 1971, an example of a modern viral vaccine success is the MMR (mumps, measles and rubella) vaccine (Larson et al., 2018). This live-attenuated vaccine contains variants of all three pathogens. MMR can be transported in lyophilized form and is usually administered subcutaneously. Developing vaccines for other viral pathogens remains challenging despite enormous technological advancements. This pertains particularly to HIV, where in spite of more than 30 years of research, no reports of a completely effective prophylactic or therapeutic vaccine exist (Dangeti, 2014). The lack of efficacy of candidate HIV vaccines has been attributed to HIV escape from vaccine specific CD8+ T cells, lack of CD8+ T cells replenishment and inability to target latently infected cells (reviewed by Perreau et al., 2017). Presently, it is accepted that a successful HIV vaccine would comprise of an optimized vector and/or immunogen proficient in stimulating broad, long-lived and safe immune responses (Esparza and Van Regenmortel, 2014). Up until 2016 only 6 HIV-vaccine candidates reached clinical trials and most of these failed to produce protective broadly neutralizing antibodies thus changing the focus of vaccine strategies to elicit CTL responses (Stephenson et al., 2016). Effective T cell-based vaccine can limit the initial viral replication and lower viral load set point, thereby preventing the initial infection or/and slow disease progression (Haynes, 2015). The first CTL based vaccine trial was the step study (HVTN502) which also did not show any protection and actually induced higher infection rates in a subset of participants (Cao et al., 1995; Duerr et al., 2012). Eventually, the RV144 trial, which used a prime-boost strategy with a canarypox viral vector and envelope protein gp120 demonstrated a 31% efficacy rate (Rerks-Ngarm et al., 2009). Results from the RV144 trail demonstrated that even non-neutralizing antibodies which match the infecting strain also have the ability to prevent infection (NCT01435135) primarily via ADCC and Fc mediated responses. The major concern with such a vaccine strategy is the enormous diversity of HIV-1 strains (Stephenson et al., 2016). To improve on the RV144 results, current strategies are focused on understanding immune correlates of protection that would increase the breadth of ADCC and FcR activities (Haynes, 2015). Although these trials have been mostly disappointing, they are informative and highlight the remarkable struggle associated in developing effective HIV vaccines and provide essential information to support pre-clinical development of other novel T cell vaccine strategies.

Unlike viral-targeted vaccines, cancer vaccines do not prevent disease but rather stimulate the immune system to attack an already existing disease. Many cancer vaccines consist of cancer cells, parts of cells, or pure antigens. Often, a patient’s immune cells are isolated and exposed to cancer antigens, and once activated, these immune cells are re-introduced into the patient’s body and are better able to suppress cancer cells. Sipuleucel-T (Provenge^®^) (Sip-T) is the first FDA (United States Food and Drug Administration)-approved therapeutic cancer vaccine. It is an autologous vaccine used for the treatment of patients with asymptomatic or minimally symptomatic castration-resistant metastatic prostate cancer (mCRPC) and who are not responsive to hormone therapy (Cheever and Higano, 2011). Sip-T elicit anti-tumor activity via activation of T cells that are specific for prostatic acid phosphatase (PAP), an enzyme found on the surface of 95% of prostate cancer cells (Alteri et al., 2018). The vaccine is formulated to comprise of a patient’s mature antigen-presenting cells (APCs) expressing PAP after *ex vivo* exposure to a granulocyte macrophage colony stimulating factor (GM-CSF)-and PAP fusion protein (Gardner et al., 2012).

There is still no clinically approved vaccine for fungal infections; however, there are a growing number of candidates in pre-clinical development and at various phases of clinical trials (Health, 2012). Fungal vaccine strategies have mainly prioritized CD4+ T cell and B cell stimulation, thereby enhancing protection mediated by these defense mechanisms (Nanjappa and Klein, 2014). This involves targeting common antigens that are shared among a variety of medically relevant fungi. One example is the β-1,3-D-glucan, a key component of the fungal cell wall (Armstrong-James et al., 2017). Mice immunized with this glucan, conjugated to diphtheria toxin, elicit strong antibody responses that are protective against models of aspergillosis, candidiasis and cryptococcosis. Moreover, immunizing mice with antigen encapsulated in glucan, also stimulate antigen-specific antibody and T cell responses. Preclinical studies involving the vaccination of mice with an attenuated strain of *Blastomyces dermatitidis* showed protection against subsequent challenge from virulent strains (Wüthrich et al., 2003). Even upon CD4+ T cell depletion, protection was seen due to the emergence of protective CD8+ T cells. More recently, the focus of fungal vaccines has been on subunit vaccines and the two containing *Candida albicans*-derived proteins, currently in phase I/II clinical trials, were found to confer immunogenicity (De Bernardis et al., 2012; Schmidt et al., 2012).

Majority of FDA approved vaccines work by stimulating a CD4+ T cell dependent antibody response. However, for cancer and intracellular infections a CD8+ cytotoxic T cell (CTL) response is required for protection. Thus far, the primary limitation of such vaccines has been a lack of ability to prime and activate disease specific-CD8+ T cells necessary for protection. This is attributed to the inherent mechanism by which external foreign antigens administered in a vaccine are presented to the immune system via MHCII, thus stimulating CD4+ based responses. Recently, these challenges have been addressed by photochemical internalization (PCI) which has been shown to deliver antigenic peptides to the cytosol of antigen presenting cells by disruption of the endosomal membranes using a co-endocytosed photosensitizer (Haug et al., 2018). The 30-fold increase in MHCI presentation and resulting 30–100-fold increase in disease specific CD8+ activation in comparison to antigen stimulation alone, is promising. Furthermore, preclinical *in vivo* experiments involving the induction of antigen-specific CTL responses against cancer antigens in mice confirmed the efficacy of PCI as a peptide-based vaccine. Strategies such as these are not only applicable to cancer by have great potential to improve various peptide vaccines especially for diseases like HIV where an appropriate CTL response is required for protection.

### Enhancing T Cell Activation

Successful T cell activation requires two signals: T cell receptor (TCR) binding to peptide-MHC complex and binding of T cell co-receptors with counter-receptors on APCs. T cell exhaustion is a state of T cell dysfunction that arises during persistent antigen exposure and/or inflammation and is associated with many chronic infections and cancer. It is characterized by persistent expression and diversity of inhibitory receptors, progressive and hierarchical loss of effector cytokines, metabolic imbalances, altered expression and function of transcription factors, failure to convert to quiescence and inability to acquire antigen-independent memory T cell homeostasis (Wherry, 2011; Schietinger and Greenberg, 2014). Thus, T cell exhaustion is a mechanism of immune evasion essentially leading to the inefficient control of infection and tumors. Importantly, exhausted T cells are not inert but sustain suboptimal, essential functions that encumber ongoing pathogen infection or tumor progression (Wherry and Kurachi, 2015).

This state of T cell dysfunction was initially described in the murine lymphocytic choriomeningitis virus (LCMV) model (Zajac et al., 1998), and has since been observed in animal and human models during chronic viral infections such as HIV (Kaufmann et al., 2007), Hepatitis C virus (HCV), Hepatitis B virus (HBV) (Guidotti and Chisari, 2006), simian immunodeficiency virus SIV (Zeng et al., 2011), along with various cancers (Lee et al., 1999), malaria infections (Illingworth et al., 2013) and *Mycobacterium tuberculosis* infection (Khan et al., 2017). Major advances have been made in three significant areas including inhibitory receptors and negative regulatory pathways, the absence of canonical memory T cell properties and maintenance, and the origin and homeostasis of exhausted T cells (Kim and Ahmed, 2010; Paley et al., 2012; Crawford et al., 2014). As such, there has been considerable interest in avoiding or reversing this dysfunctional state of exhaustion, and consequently, restoring or augmenting immune responses to effectively control infection or malignancies (Pauken and Wherry, 2015).

#### Modulating Intrinsic Inhibitory Receptors

To minimize tissue damage, negative pathways of immunoregulation such as those used in immune checkpoint inhibition offer a plethora of inhibitory pathways that are essential for preserving self-tolerance and moderating the duration and magnitude of immune responses in peripheral tissues (Pardoll, 2012). These pathways are inherently involved in T cell exhaustion and involve cell surface inhibitory receptors that modulate autoreactivity and immunopathology (Sharpe et al., 2007). Inhibitory receptors are transiently expressed in functional effector T cells, however, increased and prolonged expression can be considered a hallmark of exhausted T cells (Wherry, 2011). Pathogens and tumors promote immune checkpoint-mediated inhibitory interactions to escape immune control. Many of these checkpoint proteins have already been studied extensively and will be addressed in relation to cancer, HIV, TB, and malaria infections.

Within cancer immunotherapy, cytotoxic T lymphocyte antigen-4 (CTLA-4) and programmed cell death protein 1 (PD-1) are the canonical immune-checkpoint receptors that illustrate ligand–receptor interactions between T cells and APCs that modulate the T cell response to antigen (MHC- mediated molecule complexes that are recognized by the TCR). CTLA-4 is a co-inhibitory receptor expressed on T cells that competes with CD28, a T cell co-stimulatory receptor, for binding on shared identical ligands CD80 (B7.1) and CD86 (B7.2) (Pardoll, 2012). Binding of CTLA-4 inhibits the activation of T cells, whereas binding of CD28 to CD80/CD86 activates the T cell responses (Dyck and Mills, 2017). CTLA-4 expression on activated T cells reduces CD28 co-stimulation by; outcompeting CD28 for binding to B.7 ligands as it has a greater affinity for the B7 ligands and, possibly also by depletion of CD80/CD86 via *trans*-endocytosis (Qureshi et al., 2011). Subsequently, CTLA-4 controls the activation state of effector T cells and initiates tolerance by inhibiting cytokine production and activating transcription factor STAT5 (Srahna et al., 2006). Furthermore, CTLA-4 plays a physiological role in the negative modulation of helper T cell activity, stimulating the suppressive function of T regulatory (Treg) cells (Topalian et al., 2015).

PD-1 has also been extensively studied and predominantly modulates effector T cell activity within tissue and tumors and is therefore more widely expressed than CTLA-4; it is induced on activated T cells, B cells, macrophages, dendritic cells and Tregs. The potent inhibitory signal is provided through its interaction with programmed death-ligand 1 (PD-L1) and/or PD-L2, whereby PD-1 inhibits kinases involved in T cell activation (Latchman et al., 2001; Francisco et al., 2009; Araki et al., 2013).

CTLA-4 is constitutively expressed on all cancer cells but found to be highly expressed on breast tumor cells and is implicated in immune dysregulation and associated with poor prognosis (Mao et al., 2010; Yu et al., 2015; Yuan et al., 2015). PD-L1 is expressed on a majority of tumor cells, and tumor-derived APCs, signifying the principal role of the PD-1 pathway in tumor immune evasion associated with poor prognosis (Hirano et al., 2005; Currie et al., 2009). There is gathering evidence that inhibitory receptors have a significant role in modulating T cell responses during chronic viral infections, including HIV, as well as during acute phase malarial infections (Trautmann et al., 2006; Kaufmann et al., 2007; Crawford and Wherry, 2009; Gonçalves-Lopes et al., 2016). Furthermore, CTLA-4 is expressed on Tregs and CD8+ T cells, while PD-1 is expressed on neutrophils, Tregs and natural killer (NK) cells of patients infected with *M. tuberculosis*, however, over a course of standard anti-TB therapy, this expression declines as responsiveness of effector T cells to antigenic stimulation improves (Merlo et al., 2001; Li et al., 2015; Zumla et al., 2016a).

Lymphocyte activation gene-3 (LAG-3), T-cell immunoglobulin-3 (TIM-3), band T lymphocyte attenuator (BTLA) and T-cell immunoglobulin and ITIM domain (TIGIT) are highly expressed on exhausted T-cells and represent the next generation of immune checkpoints that are under evaluation for the treatment of chronic diseases including cancer and chronic viral infections such as HIV (Topalian et al., 2015; Chew et al., 2016; Dyck and Mills, 2017; Grabmeier-Pfistershammer et al., 2017; Manieri et al., 2017). Interestingly, Anderson et al. (2016) recently proposed a model describing LAG-3, TIM-3, and TGIT as second tier co-inhibitory molecules that play a specific role in regulating immune responses at sites of tissue inflammation, distinct to first tier co-inhibitory receptors CTLA-4 ad PD-1 which are primarily involved in maintaining self-tolerance. This hierarchal ranking of co-inhibitory receptors provides a basis for selecting synergistic receptor blockade combinations that will result in improved T cell and NK cell effector function (Anderson et al., 2016).

##### Immune checkpoint inhibitors

As immune checkpoints are mediated by ligand–receptor connections, these pathways can be easily blocked by monoclonal antibodies (mAbs) or regulated by recombinant forms of ligands or receptors (Pardoll, 2012). As such, PD-1 and CTLA-4 present ideal targets for immune checkpoint inhibition therapies, one of the most noteworthy immunomodulatory therapies of current times. In contrast to vaccines which activate immune cells to attack specific targets, this type of therapy removes the natural disruptions of the immune system (Postow et al., 2015).

Subsequent to the clinical success of Ipilimumab, an anti-CTLA4 mAb for the treatment of advanced melanoma which acts by blocking the binding of CTLA-4 expressed on T cells to CD80/86 ligands expressed on tumor cells (Kyi and Postow, 2014), an onslaught of immune checkpoint inhibitors have received accelerated FDA-approval. This includes Pembrolizumab and Nivolumab, both recently approved mAbs which bind the inhibitory receptor PD-1, blocking its interaction with ligands PDL-1/PDL-2, thus releasing T cells for cytotoxic action (Nguyen and Ohashi, 2015). These immune-checkpoint inhibitors are effective for the treatment of melanoma, renal cell carcinoma, and non-small lung cancer (Robert et al., 2015a,b). In addition to targeting inhibitory receptors, blockade of the ligand PD-L1, by Atezolizumab, Durvalumab, and Avelumab, has demonstrated remarkable efficacy in lung, bladder, urothelial carcinoma and other cancers (Massard et al., 2016; Balar et al., 2017; Hsu et al., 2017; Rittmeyer et al., 2017).

However, most patients do not respond to immune checkpoint monotherapy; attributed to the absence of tumor-infiltrating effector T cells and tumor microenvironment properties. As checkpoint inhibitors target ligands/receptors of immune regulation, expression of these targets on target cell membranes is an essential prerequisite for the treatment to be effective. More than 50% of cancers induce a quiescent immune tumor microenvironment which is naturally depleted of immune effector cells and thus, lacks sufficient targets for checkpoint immunotherapy to be effective. Recently, the high levels of circulating exosomal PD-L1 was shown to correlate with immune dysfunction, suggests a role as a predictive biomarker for patient responses to anti-PD-1 therapy (Chen et al., 2018). Furthermore, combining checkpoint therapies with cancer vaccine strategies like PCI has the potential to significantly enhance the efficacy of both therapies. Cancer vaccines stimulate CD8+ T cells which express checkpoint targets; consequently the normal inhibitory effects of checkpoints would be reduced by checkpoint therapy, thereby further enhancing CD8+ activation. The synergistic effects of cancer vaccines and checkpoint combination therapy has been demonstrated (Duraiswamy et al., 2013; Karyampudi et al., 2014; Fu et al., 2015; Soares et al., 2015). The positive pre-clinical results of some of these studies has initiated 2 clinical trials assessing various vaccines in combination with nivolumab for the treatment of pancreatic cancer (Kleponis et al., 2015).

In addition to cancer vaccines, combinations of other immunotherapies with checkpoint inhibitors, holds promise for the rational development of curative therapies. The simultaneous blocking of the CTLA-4 and PD-1 pathways, given their distinct and non-overlapping mechanisms of action, has been the most successful combination tested thus far, resulting in tumor regression in patients with advanced melanoma, demonstrating the synergistic effects of combining checkpoint inhibitors (Wolchok et al., 2013; Hodi et al., 2016; Weber et al., 2016). The safety and efficacy of various combinations of immune checkpoint inhibitors with cytotoxic chemotherapy (referred to as immunochemotherapy), with small-molecule inhibitors or with radiation therapy are also under intense clinical investigation^1^ (Mahoney et al., 2015; Li et al., 2017). Recently, prolonged progression-free survival of patients with metastatic triple-negative breast cancer treated with a combination of the chemotherapy drug nab-paclitaxel and the PDL-1 inhibitor Atezolizumab, was demonstrated in a Phase 3 trial (Schmid et al., 2018).

The development of primary or acquired resistance remains clinically pertinent. Recent studies suggest that inactivation of JAK1 and JAK2 by cancer cells results in resistance to interferon gamma which contributes to checkpoint resistance and immune escape. In addition, resistance is likely driven by multiple other mechanisms including loss of function of CD8+ T cells due to a lack of mutational or shared antigens required for T cell recognition, reduced antigen presenting components, tumor cell induced T-cell signaling inhibition and depressed sensitivity to apoptotic inducting molecules (Shin et al., 2016; Zaretsky et al., 2016). Methods of reducing these types of resistances would need to be considered for the development of new checkpoint therapies.

**FIGURE 2:**
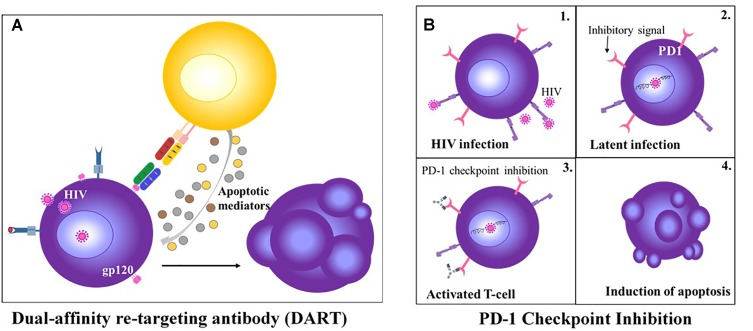
HIV-targeting T cell Therapies. **(A)** Anti-CD3 and anti-gp120 DART treatment redirects CD8+ T cell to kill HIV infected CD4+ T cells (Perreau et al., 2017). **(B)** PD-1 check point inhibition of latently infected CD4+ T cell results in re-activation of the T cell and induction of apoptosis (Wykes and Lewin, 2018).

In addition, as with other therapies, immune checkpoint therapies are associated with a high rate of toxicities, characterized as immune-related adverse events (IRAEs) and include diarrhea/colitis and hepatic/dermatologic/endocrine toxicities. These adverse effects, although severe, are manageable with immunosuppressive agents such as corticosteroids, which in turn, may also be associated with other short-term side effects (Michot et al., 2016; Spain et al., 2016). In chronic infection, the preclinical development of checkpoint inhibitors has had mixed results, suggesting intrinsic differences in the mechanisms of T cell dysfunction between chronic infection and cancer. In *in vitro* settings, CTLA-4 blockade enhanced HIV-specific CD4+ T cell function, PD-1/PD-L1 blockade improved HIV-, HCV-, and HBV-specific CD8+ T cell function, while CTLA-4/PD-1 combination blockade reversed HCV-specific CD8+ T cell exhaustion (Kim and Ahmed, 2010). However, in chronic LCMV-infected mice, neither CTLA-4 nor LAG-3 blockade improved T cell function or viral load, whereas PD-1 blockade alone and in combination with LAG-3 blockade effectively salvaged T cells from exhaustion and decreased the virus load (Barber et al., 2006). In the SIV macaque model, CTLA-4 blockade did not enhance viral-specific T cell responses, but rather amplified viral replication at mucosal sites, whereas a recent study demonstrated anti-PD-1 treatment delayed viral rebound after combined antiretroviral therapy (cART) was withdrawn (Cecchinato et al., 2008; Gill et al., 2016). The *ex vivo* dual blockade of co-inhibitory receptor TIGIT and ligand PD-L1 restored HIV-specific CD8+ T cell effector responses (Chew et al., 2016). In HIV-infected patients, treatment with Ipilimumab resulted in reactivation of latently virus-infected CD4+ T-cells, rendering these cells discernable to the immune system (Wightman et al., 2015) (Figure 2). Furthermore, a recent Phase I trial demonstrated enhanced HIV-specific CD8+ T cell responses following treatment with an anti-PD-L1 antibody (Gay et al., 2017). Overall, these studies suggest a role for immune checkpoints in limiting T cell responses during HIV infection and the subsequent potential of immune checkpoint inhibitors. Although the anti-PD-1 therapy Nivolumab effectively reduced viral load in HCV-infected individuals, it has been proposed that established virus-specific T cells in the liver is crucial for enhanced T cell responses by PD-1 blockade (Fuller et al., 2013; Gardiner et al., 2013). In patients with pulmonary TB, PD-L1 is highly expressed on *M. tuberculosis*-infected macrophages, and *in vitro* blockade with mAbs induces IFN-γ-initiated killing of infected cells by autologous peripheral blood T cells (Zumla et al., 2016a).

While checkpoint inhibitors have revolutionized cancer immunotherapy there are clearly also many drawbacks associated with this therapy at this stage. The many combination immunotherapy studies involving checkpoint inhibitors will provide much needed information on the molecular mechanisms of resistances and toxicity associated with checkpoint therapy and subsequently assist in improving efficacy. Despite the many issues that still require addressing there have been overwhelming positive results associated with PD1/PDL-1 and CTLA-4 checkpoint inhibition studies demonstrating the potential of checkpoint inhibitors to greatly improve treatment outcomes not only in cancer patients but with other diseases as well, especially in combination with other immunotherapies.

#### Cytokine Therapy

Cytokines are protein mediators involved in essentially all important biological processes including immunity, cell proliferation and inflammation, wound healing and repair, cell migration, fibrosis and angiogenesis. Cytokines provide crucial signals for fundamental processes involved in a plethora of diseases thus it is intuitive that manipulation of cytokines may alter diseased states both positively and negatively. With the field of cancer, multiple mice studies have demonstrated the anti-tumor effects of cytokines leading to cytokine-based therapies for cancer treatment. Cytokines such as IL-1, IL-12, IL-15, IL-18, IL-21, and GM-CSF are just some examples of cytokines that have entered clinical trials for treatment of patients with advanced cancer (Feldmann, 2008). Recently, administration of pro-inflammatory cytokines IL-12 and IL-18 demonstrated a reversal in MHC-1 deficient tumor induced NK cell anergy. Treatment increase NK cell activity which mediated antitumor responses resulting in improved survival of mice challenged with MHC-1 deficient tumors (Ardolino and Raulet, 2016).

Neutralization of suppressive cytokines like TGF-β and IL-10 have shown promising results and will the remain the focus of this section. IL-10 is expressed by diverse cell types such as dendritic cells (DCs), monocytes and/or CD4+ T cells, and its upregulation is associated with disease progression during chronic infections as well as poor prognosis in cancer patients (Said et al., 2010; Richter K. et al., 2013). Furthermore, IL-10 has been implicated in promoting human visceral leishmaniasis, by restricting Th1 cell-type responses and/or neutralizing parasitized tissue macrophages and, in parallel, compromising responsiveness to chemotherapy (Murray et al., 2002). During *in vivo M. tuberculosis* infection, IL-10 has been shown to be a negative regulator of the immune response, contributing to chronic infection (Pitt et al., 2012). In addition, fungal clearance is impaired by IL-10, as demonstrated by *Cryptococcus neoformans* infections, the leading cause of fatal mycosis in HIV+ individuals (Murdock et al., 2014).

Blockade of the IL-10 receptor (IL-10R) with anti-IL-10 mAbs enhances control of LCMV infection and restores T cell responses (Brooks et al., 2006; Ejrnaes et al., 2006), while simultaneous blockade of IL-10 and PD-1 reverses CD8+ T cell exhaustion, improving HIV control (Wherry, 2011). Furthermore, simultaneous IL-10 blockade during therapeutic DNA vaccination enhanced clearance of chronic LCMV infection (Ni et al., 2015). In the mouse model, the use of antimony, the conventional chemotherapy for visceral leishmaniasis, in conjunction with anti-IL-10R treatment permitted a considerable reduction in duration and dosage of treatment (Weiner et al., 2012). During *M. tuberculosis* infection, antibody blockade of IL-10R specifically during BCG vaccination improves Th1 and Th17 responses, and IFN-γ and IL-17A expression *in vivo*, resulting in significantly enhanced protection against aerogenic challenge with *M. tuberculosis*, compared to BCG vaccination alone (Pitt et al., 2012). In the murine model for *C. neoformans* infection, IL-10 blockade amplified various effector mechanisms, including improved accumulation of CD4+ T cells, B cells, Th1 and Th17 cells, but not CD8+ T cells, and, furthermore, effectively abrogated dissemination of infection to the brain (Murdock et al., 2014).

TGFβ is often over-expressed in several tumors and has been implicated in promoting growth, progression, and metastatic potential of advanced cancers (Jakowlew, 2006; Tian et al., 2011; Drabsch and Ten Dijke, 2012). Various TGFβ pathway inhibitors including small molecules, anti-TGFβ antibodies and synthetic small peptides have been reported to restore immune response and increase the efficacy of combination immunotherapy (Neuzillet et al., 2015). During chronic viral infection, both murine and human, TGFβ signaling has been implicated in T cell exhaustion (Wherry and Kurachi, 2015). However, in contrast to cancer therapy, the systemic administration of antibody- and inhibitor-based strategies to prevent TGFβ signaling during chronic viral infection have proven futile, at least in the murine model, the reasons for which remain unclear (Boettler et al., 2012; Garidou et al., 2012).

Given that cytokines play such a multifunctional role in different biological systems, systemic inhibition of any given cytokine will have wanted and unwanted effects, necessitating the need to make this therapy more targeted. Cytokines have differing signaling cascades that may be exploited for selecting a target to inhibit their functions. TNF is an ideal example; its two forms, membrane-bound or secreted, each interact with distinct receptors, TNFR1 or TNFR2, differentially expressed on multiple cell types (Drutskaya et al., 2018). Depending on the source of cells secreting TNF, and the neighboring cytokines, it may act as pro-inflammatory or anti-inflammatory. Thus, targeting selected downstream pathways may provide a more controlled treatment option than systemic TFN inhibition. TNFR1 is responsible for majority of TNF pro-inflammatory functions which signals via NF-κB and AP-1 resulting in expression of other pro-inflammatory cytokines like IL-1 and IL-6 (Simmonds and Foxwell, 2008). A TNFR1-specific antibody, ASTROSAB has demonstrated effective inhibition of TNF induced IL-6 and IL-8 secretion in cell culture (Richter F. et al., 2013). A second study in chimeric humanized mice demonstrated that the inhibition if TNFR1 by a human TFNR1 silencer (TROS) lowered TNFR1-induced proinflammation reactions but did not alter TNFR2 signaling (Steeland et al., 2015). Studies looking at the source of TNF found that TNF produced by myeloid cells play a role in pathogenesis of experimental arthritis and autoimmune encephalomyelitis (Grivennikov et al., 2005; Kruglov et al., 2011). Contrastingly TNF secreted by T cells demonstrated protection against *M. tuberculosis* infections (Allie et al., 2013). TNF perfectly demonstrates the pleiotropic functionality typical of pro-inflammatory cytokines, balancing regulatory functions and pro-inflammatory signaling to maintain homeostasis of the immune system. Thus, systemic knock-out of TNF while beneficial in controlling certain inflammatory and autoimmune diseases, has major drawbacks like increased risk of severe infections, cardiovascular, neurological and immunological complications. Currently antibody-based immunotherapies with dual affinities for TNF and specific cell types are in development; one example is a myeloid-specific TFN inhibitor (MYSTI) that binds to both myeloid cells and TNF thus only restricting myeloid-secreted TNF activity. MYSTI demonstrated better protection against LPS/D-gal hepatotoxicity in humanized mice compared to anti-TNF treatment (Efimov et al., 2016).

Multiple other recombinant cytokines have already been approved for treatment of patients; IL-2 for cancer (Baron and Narula, 1990; Dutcher, 2002), several IFNα derivatives for cancer and viral infections (Syed and Ahmadpour, 1998; Melian and Plosker, 2001; Marcellin et al., 2004), IL-11 thrombocytopenia induced by chemotherapy (Isaacs et al., 1997), IFNβ for multiple sclerosis and IFNγ for osteoporosis and cancer (Cutler and Brombacher, 2005). Understanding the molecular mechanisms of action of different cytokines in the context of a specific disease will be an essential prerequisite for developing more targeted approaches to anti-cytokine/cytokine therapy. Targeting specialized processes rather than systemic cytokines has the added benefit of restricting toxicity and reducing interference with non-related biological processes. With the exponential development in antibody technologies it is likely that cytokine therapies in the near future might all be targeted to specific functions rather than cytokines overall.

### T Cell Engineering

#### Recruitment and Activation of Cytotoxic T Cells

Bispecific antibodies (BsAbs) have two binding sites, each with a unique antigen specificity. Multiple formats of bsAbs exist including bi-specific T cell engagers (BiTEs), dual-affinity re-targeting antibodies (DARTs) and Tandem Diabodies. The most commonly used format is a tandem scFv, of which BiTEs are the most clinically advanced in oncology and are distinguished for their binding specificity to the TCR complex. These antibodies comprise both an antigen-specific arm and a T cell activating arm via generic CD3-interaction, which bypasses the requirement for MHC-I-restricted engagement. The activated T cells are redirected to target cells through the recognition of a pathogen/tumor-specific antigen by BiTE antibodies (Wolf et al., 2005; Bargou et al., 2008). BsAb mechanism of action is via creation of a temporary cytolytic synapse between the T cell and the target cell and the ensuing activation and proliferation of T cells elicits target cell lysis (Offner et al., 2006). The immunological synapses created by BiTEs are indistinguishable from those induced during natural CTL responses. The size of the synapse is determined by the distance between anti-CD3 and anti-antigen moieties and is a vital measure of cytotoxic potency; the smaller the synapse the closer the T cell membrane is to the target cell and since delivery of apoptotic mediators, such as granzyme proteases and perforin, is conducted by passive diffusion, this results in more efficient target-cell lysis (Moore et al., 2011).

BsAbs have been posited for the treatment of malignancies and inflammatory conditions for decades but have only recently begun to yield results (Fan et al., 2015). As such, catumaxomab (Removab) was the first bispecific trifunctional drug approved for the treatment of malignant ascites (Linke et al., 2010; Sedykh et al., 2018). Thereafter, blinatumomab (Blincyto^®^), a CD19 × CD3 BiTE, gained accelerated FDA-approval for the treatment of relapsed and/or refractory (R/R) acute lymphoblastic leukemia (ALL), and numerous other BiTE candidates are currently in phase I clinical trials for the treatment of various cancers (Przepiorka et al., 2015). Furthermore, as T cell exhaustion due to high antigen load has been suggested, concurrent PD-1 or PD-L1/2 blockade is under investigation (Köhnke et al., 2015). Studies are now focused on the efficacy of BiTEs for solid tumor malignancies, in which penetration of the tumor by BiTEs and T cells are at the forefront (Yuraszeck et al., 2017).

Given the success of bsAbs in cancer immunotherapy, similar strategies to eliminate pathogen-infected cells have been adopted. Following early studies on natural HIV receptor CD4 and an anti-CD3 binding moiety, no further development of bsAbs ensued for more than 20 years (Berg et al., 1991). Recently, DARTs, an alternative tandem ligand format, have been exploited for T cell–mediated cytolysis of latently HIV-infected cells (Figure 2). These bsAbs comprise anti-Env and anti-CD3 arms which simultaneously bind to HIV-infected cells and CD3-expressing polyclonal T cells, subsequently inducing CD8+ T cell elimination of HIV-infected CD4+ T cells *in vitro*, reducing virion levels *ex vivo* and mediating clearance of latent HIV reservoirs from resting CD4+ T cells (Sloan et al., 2015; Sung et al., 2015). The efficacy of DARTs on the HIV-1 reservoir *in vivo* remains undetermined. Apart from DARTs, BiTEs that target the HIV-1 envelope protein gp120 and CD4 have been recently described with potent antiviral activity *in vitro* and *ex vivo* (Brozy et al., 2018). In addition to HIV, bsABs have been developed to redirect effector T cells to HBV- and human cytomegalovirus (HCMV)-infected cells, *in vitro* (Meng et al., 2018).

The appeal of BsAbs lies in their synergistic effects attributed to their ability to simultaneously target several antigens (Sedykh et al., 2018). Although a promising therapeutic strategy, BsAbs are not without their challenges. In clinical settings, continuous delivery is necessary due to the rapid clearance of these agents, related to their small size and consequently, short half-life. Furthermore, blinatumomab is associated with severe adverse events including neurotoxicity and cytokine release syndrome, which although reversible and manageable by treatment with corticosteroids and/or Tocilizumab, an interleukin-6 receptor antagonist, discontinuation of use by patients is still common (Thakur et al., 2018). In addition, clinical success in solid tumors remains to be demonstrated, which may provide insights into the applicability of these agents against intracellular pathogens.

#### Reprogramming T Cells

Chimeric antigen receptor (CAR) T cells are modified T cells with a recombinant receptor, comprising a scFv that redirects the specificity of effector T-lymphocytes, fused to a transmembrane and signaling domain that mediates T cell activation without MHC engagement. By bypassing the standard kinetics of active immunization, CARs rapidly generate tumor-targeting T cells and modulate T cell proliferation and persistence in the tumor microenvironment (Ho et al., 2003; Sadelain et al., 2003). The therapy involves the *ex vivo* expansion of the patient’s peripheral blood T cells, genetically modifying the cells to express the CAR and re-infusing the engineered T cells back into the patient (Gattinoni et al., 2006).

First generation CAR T cells comprising a targeting scFv and chimeric CD3/FcεRIγ have undergone phase I clinical trials for the treatments of ovarian cancer (scFv-FcεRIγ) (Sadelain et al., 2003), refractory follicular lymphoma (scFv-CD3ζ) (Ho et al., 2003), neuroblastoma and melanoma (scFv-CD3ζ) (NCT00085930) (Zhao et al., 2006; Birkholz et al., 2009). In theory, any cell surface molecule, even those that elicit tolerance due to self-reactivity, may be targeted by CAR modified T cells, thereby increasing the natural T cell repertoire. Transgenic mice studies with first generation CARs recognized that these CAR T cells were prone to anergy (Brocker and Karjalainen, 1995). Thus, second and third generation CARs comprising an activating CD3 domain and cytoplasmic domain co-stimulatory receptors like CD28, 4-1BB, DAP10, ICOS or OX40 were generated. These CARs demonstrated superior activity as confirmed by a comparative study in patients treated with CD28/CD3ζ or CD3ζ-only CAR (Savoldo et al., 2011).

Chimeric antigen receptor T cell immunotherapy has achieved impressive clinical outcomes for the treatment of hematological malignancies, specifically B cell acute lymphoblastic leukemia (ALL) and lymphoma (Kochenderfer and Rosenberg, 2013; Maude et al., 2014). As such, two CD19-targeted CAR T cell therapies, Tisagenlecleucel (2nd generation) and Axicabtagene Ciloleucel (3rd generation) were recently FDA-approved (Neelapu et al., 2017; Schuster et al., 2017). Majority of cancer-related morbidity and mortality is associated to solid malignancies, however, attempts to apply CAR T cell therapy to solid tumors have been less effective compared to hematologic cancers. This has been attributed to a number of obstacles; the lack of suitable tumor-specific antigens and the subsequent toxicity to normal tissue, inefficient CAR T cell infiltration of tumor barriers, such as the extracellular matrix (ECM), as well as the immunosuppressive tumor microenvironment (Zhukovsky et al., 2016; Srivastava and Riddell, 2018).

As such, genetically modified fourth generation CARs also known as TRUCKs (T cell redirected for universal cytokine-mediated killing) with the ability to secret pro-inflammatory and pro-proliferative cytokines have been developed (Golubovskaya and Wu, 2016). This ability to induce cytokine expression is highly advantageous in the setting of solid tumors where T-cell penetration is poor and due to high phenotypic diversity, many cancer cells remain invisible to penetrating T-cells. The expression of cytokines such as IL-12 induced by TRUCKs provides a cytokine gradient and initiates universal cytokine mediated killing of cancer cells which eradicates cancer cells lacking antigen at the tumor site (Li et al., 2018). Recently, a TRUCK system utilizing the synNotch receptor was developed to express a variety of payloads in response to target antigens (Roybal et al., 2016; Roybal and Lim, 2017). In addition to inducible cytokines this synNotch system can induce adjuvants, checkpoint, bispecific and pro-tumor cytokine antibodies. With this system more anti-tumor cytokines and factors with the potential to remodel the suppressive tumor environment may be co-administered.

The unique obstacles imposed by solid tumors are also associated with brain tumors, although further exacerbated by additional biological complexities. With regards to glioblastoma, the most common malignant brain tumor in adults, recent developments in CAR T cells have addressed these challenges which include penetration through the blood–brain barrier (BBB), inadequate neoantigen presentation and the presence of glioma stem cells (GSCs) and their intrinsic role in drug and radiation resistance [reviewed by (Bagley et al., 2018)]. The poor homing of CAR T cells to target sites (Ager et al., 2016) has recently been addressed by the rational reengineering of CD6 to generate cytotoxic homing system (HS) T cells that readily infiltrate the otherwise restrictive BBB (Samaha et al., 2018). Studies such as these may provide significant insight into engineering T cells to target elusive infectious agents.

While most CAR T cell studies conducted to date are in the context of cancer immunotherapy, Kumaresan and colleagues developed a fungal-specific Dectin-1 C-type lectin receptor. The resulting D-CAR T cell specific for β-1.3-D-glucan showed effective killing of *Aspergillus fumigatus in vitro* and *in vivo* (Kumaresan et al., 2014). This proof-of-principle study shows that CAR technology can encompass C-type lectins, a key innate pattern recognition receptor with broad repertoires against fungal pathogens and thus, may be applied to other infectious diseases (Armstrong-James et al., 2017).

As with other immunotherapies, adverse effects are associated with CAR T cells. These include the on-target toxic effects of cytokine release syndrome (CRS) and neurotoxicity which can be managed by treatment with corticosteroids and/or Tocilizumab (Gauthier and Turtle, 2018). In a recent phase I/II clinical trial, CRS severity was found to be associated with infectious complications (Hill et al., 2018). Reducing the adverse effects of treatment, improved models to evaluate pre-clinical efficacy and importantly, the ultimate cost implications to patients, will determine the extent to which CAR T cell therapy can be applied (Srivastava and Riddell, 2018). The advances in CAR T cell therapy for solid tumors offers promise for intracellular infectious agents, such as *M. tuberculosis*, for which the inherent heterogeneity of granulomas accounts for poor antimicrobial drug penetration and accumulation (Kiran et al., 2016).

#### Depletion of Suppressor Cell Populations

Tregs are a subpopulation of T cells that modulate the immune system by reducing activation of effector T cell populations during prolonged inflammatory responses and inducing tolerance to benign molecules and self-antigens. Modern cancer immunotherapy approaches include biologics that selectively deplete suppressor cell populations, like Tregs. One such agent, a diphtheria toxin (DT) (Liedtke et al., 2008)-related interleukin 2 (IL-2) fusion protein, known as DD depletes cells with high expression levels of CD25 (Kiyokawa et al., 1991). DD is comprised of 3 domains; the DT catalytic domain, the DT membrane translocation domain and a substituted human IL-2 instead of the native DT-receptor. The IL-2 moiety allows for selective targeting of IL-2R bearing cells. Upon IL-2 and IL-2R binding, the fusion protein is internalized and transported to the cell cytosol where the catalytic domain inhibits protein synthesis resulting in downstream alterations that ultimately lead to apoptosis (Zahaf and Schmidt, 2017). DD has been approved for treatment of cutaneous T cell lymphoma and subsequent human studies. Results from these studies indicate that in addition to killing CD25+ lymphoma T cells, DD also transiently depleted Tregs (Litzinger et al., 2007). More recent studies assessing DD treatment in Leishmanian-infected mice verified a reduction of lesional Tregs, better lesion resolution and reduced parasite burden (Divanovic et al., 2011).

During *M. tuberculosis* infection, bacilli are engulfed by dendritic cells in the lung and transported to lymph nodes where CD4+ and CD8+ T cells are activated. Tregs have an opposing effect that limit potential protective responses and facilitate bacterial replication (Kursar et al., 2007). Additional studies using specific antibodies to deplete Tregs in *M. tuberculosis*-infected mice showed decreased bacterial burden (Gupta et al., 2017). Similarly to Tregs, another suppressor cell type, myeloid-derived suppressor cell (MDSC) populations also increase during *M. tuberculosis* infection contributing to T cell dysfunction and exuberant inflammation (Knaul et al., 2014). As with Tregs, specific antibody depletion of MDSCs ameliorated pathology and reduced bacterial replication *in vivo*. In addition to the suppressive effect of these cell populations, both Tregs and MDSCs express IL-2R, rendering these cell populations susceptible to DD treatment. *M. tuberculosis*-infected mice treated with DD monotherapy, or in conjunction with standard anti-TB treatment of indicated the effective reduction of Treg and MDSC populations resulting in reduced bacterial burdens (Gupta et al., 2017). While no studies have yet been conducted in the patients with TB, these studies suggest that IL-2 receptor targeting might be a viable treatment option in the future and should be validated in clinical trials.

Although the role of Tregs in the pathogenesis of HIV and HCV infections remains controversial, several studies have indicated Treg-mediated suppression of effector T cell responses in these diseases (Li et al., 2008). As such, the Treg depletion mouse model (DEREG), which expresses the diphtheria toxin (DT) receptor under the control of the *Foxp3* promoter, has shown the reactivation of virus-specific CD8+ T cells following the transient depletion of Tregs during chronic viral infection (Kim et al., 2007; Lahl et al., 2007; Dietze et al., 2011; Berod et al., 2012). The limitations to DEREG, including DT-mediated toxicity, warrants further studies to investigate the role of Tregs during chronic viral infection (Christiaansen et al., 2014).

## Antibody/Ligand-Based Therapies

### Selecting the Ideal Ligand

**FIGURE 3:**
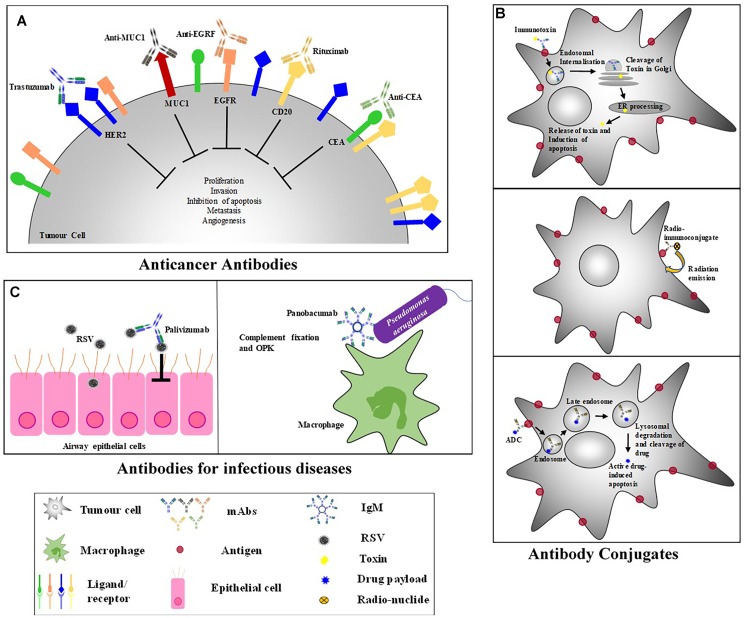
Antibody-Based Therapeutic Strategies. **(A)** Anticancer antibodies eliminate cancer cells and cause tumor destruction by targeting cancer antigens. **(B)** Antibody-conjugates – (i) Immunotoxins: bind to a surface receptor of an infected cell, undergo endocytosis and intracellular trafficking to the cytosol where most toxins induce cell death; (Becker and Benhar, 2012) (ii) ADCs: combine the specificity of mAbs with the cytotoxic potential of drugs and binds to internalizing receptors on target cell and are taken up by endocytosis; Once in the cell, ADCs undergo cellular trafficking to a lysosome where lysosomal degradation results in the cleavage and release of the active drug into the cellular cytoplasm where the drug induces apoptosis; (Scotti et al., 2015) (iii) Radioimmunoconjugates: antibodies attached to a radioactive molecule, once the antibody binds the target cell, the radio-particle’s radiation interacts with target cells, resulting in cell death. **(C)** Anti-viral antibodies – to eliminate a viral inhibition of cell infection, viral replication, cell-cell transmission, viral release as well as mediated killing of infected cells needs to occur; Palivizumab is a neutralizing antibody that binds to RSV preventing virus-host cell interactions (Groothuis and Nishida, 2002). Most antibacterial therapeutic mAbs function by inducing complement fixation and opsonophagocytic killing (OPK) of target bacteria; Panobacumab induces macrophage OPK of *Pseudomonas aeruginosa* (Que et al., 2014).

The variety of mechanisms of action that exist to accomplish ligand-mediated control of disease suppression designates this approach an attractive one. The distinct structure of mAbs/natural ligands define their highly efficient function in immunity and underpins antibody/ligand engineering and design. The aim of ligand-targeted therapeutics (LTT) is to neutralize or eliminate pathogenic infections and/or malignant cells (Figure 3). This is done by three major mechanisms of action: (a) inhibiting the function of certain molecules, (b) targeting particular cells or (c) acting as signaling molecules (Brekke and Sandlie, 2003).

The complex set of interaction that occur between antibodies/ligands and their cognate receptors, marks choosing an appropriate targeting ligand a crucial step in developing successful targeting applications. Multiple variables should be accounted; the level of receptor expression, the binding affinity of the targeting molecule (Damelin et al., 2015), a role for receptor-mediated internalization, the use of non-antibody based ligands and full antibody or antibody-fragment (Kovtun et al., 2006). For most therapies, the target antigen/receptor should ideally be expressed in high density on the target cell surface to permit accurate selectivity (Beck et al., 2017). In addition, avoidance of a high degree of target heterogenicity may limit off-target effects. Importantly, targets which are shed are not favored as free targets may compete with cell-bound targets thus reducing the therapeutic effects of a treatment (Allen, 2002).

Non-antibody ligands such as Fas, tumor necrosis factor (TNF) and TFN-related apoptosis-inducing ligand (TRIAL) are often easily available, relatively cheap to manufacture and easy to handle (Mathew and Verma, 2009). The main disadvantage is their relatively non-selective expression on diseased cells, making antibodies the preferred targeted delivery agent (Allen, 2002). Furthermore, recent advances in antibody engineering and phage display have improved the identification and selection of antibodies/antibody derivatives with a high degree of specificity and wide range of binding affinity for target tissue/cells (Zhao et al., 2016).

The use of whole antibodies is advantageous, allowing increased binding avidity that occurs through multiple binding sites on one molecule. Additionally, antibodies possess an Fc domain which engages with other immune cells that potentiate the natural immune response by antibody-dependent cellular cytotoxicity and complement-dependent cytotoxicity. Whole antibodies may also be more stable that antibody fragments over prolonged storage periods (Allen, 2002). Despite the advantages, whole antibodies also have their limitations; they commonly bind to normal tissues via Fc receptors, specifically on macrophages resulting in high liver and spleen uptake that can cause increased immunogenicity. Fab and scFv (single-chain variable fragments) lack both an Fc domain and complement-activating region thus reducing the potential for immunogenicity. Their smaller size also permits better cellular penetration which is a desirable effect of certain treatments. Furthermore, scFvs are attractive choices due to ease of identification and production, for example using phage display or *Escherichia coli* fermentation, as well as their reduced immunogenicity (Ahmad et al., 2012).

### Therapeutic Monoclonal Antibodies

Monoclonal Antibody (mAb) therapy has been studied and developed for the treatment of cancer, more so than for any other disease area. Subsequently, the trials and errors and ensuing solutions encountered in the cancer field, have paved the way and advanced antibody-based therapies. These include the initial complications associated with administering mouse hybridoma-derived antibodies to humans and the human anti-mouse antibody (HAMA) response which was subsequently resolved by chimerization and humanization, using recombinant DNA technology (Morrison et al., 1984; Jones et al., 1986). Thus, after years of research, a variety of mAb-based approaches that mediate their antitumor effects through diverse mechanisms have been developed (Weiner, 2015). Attesting to the success of these strategies, numerous mAbs have received clinical approval for the treatment of various cancers, as reviewed by (Kim and Kim, 2015).

The inhibitory activity of mAbs is conducted by preventing soluble mediators like growth factors and cytokines from reaching their target receptors. This is achieved by binding to the mediator or its cognate receptor. Targeting involves directing antibodies to a particular cell population where Fc receptor functions are elicited or to which effector moieties are transported. Subsequently, antibody signaling is conducted by cross linking of receptors related to programmed cell death or by specific receptors that function as agonists for activation of specific cell types (Brekke and Sandlie, 2003).

Antibody therapy for infectious diseases can be traced back more than 100 years ago to serum therapy for the neutralization of bacterial toxins (Graham and Ambrosino, 2015). In comparison to neutralization of toxins, prevention of viral diseases with the use of mAbs is more complex. To eliminate a viral infection a number of events need to occur, these include inhibition of: cell infection, viral replication, cell–cell transmission, viral release as well as mediated killing of infected cells (Parren and Burton, 2001; Burton, 2002). A typical immune response to such an infection results in the generation of specific polyclonal antibodies. Some of these antibodies are neutralizing others are not. The overall outcome, with a combination of blocking, neutralizing and eliminating antibodies might indeed be effective protection. The anti-RSV mAb Palivizumab is approved for high risk infants, and is clinically effective prophylactically, but not therapeutically (Hu and Robinson, 2010). As such, super-antibodies are a new generation of highly potent and/or broadly cross-reactive mAbs that offer prophylactic and therapeutic possibilities for viral infections. This includes highly antigenically variable viruses such as HIV, influenza virus, and Ebola virus, the treatments of which by super-antibodies, are under clinical evaluation (Walker and Burton, 2018).

As with most antibodies, surface antigens are attractive targets for antibacterial mAbs. The key features of surface-specific antibacterial mAbs are complement fixation for engagement of the host immune system and opsonophagocytic killing (OPK). Panobacumab, one of the most clinically advanced antibacterial mAbs, is a human anti-*Pseudomonas aeruginosa* LPS serotype O11 (Horn et al., 2010; Que et al., 2014). This mAb showed OPK activity *in vitro* and exhibited decreased bacterial burden and improved survival in infection models (Horn et al., 2010). In a phase II clinical trial, Panobacumab was used adjunctively with standard of care antibiotics resulting in shortened duration to disease resolution in *P. aeruginosa* 011 pneumonia patients (Que et al., 2014). While strain-specific mAbs have shown promising results, its activity is limited to strains that express its target receptors. Contrastingly, a protective mAb targeting *P. aeruginosa* type III secretion protein, PcrV plays an important role in transporting virulence factors into host cells (Frank et al., 2002). This led to the development of KB001, a phase II mAb for the prevention of *P. aeruginosa* ventilator-associated pneumonia (Baer et al., 2009).

In addition, the secreted virulence factor, alpha toxin (AT) which causes cellular death by the formation permeable pores in the plasma membrane, is targeted by two anti-staphylococcal mAbs currently undergoing efficacy trials (Berube and Wardenburg, 2013; Oganesyan et al., 2014). This prospective therapy has demonstrated prophylactic protection in *Staphylococcus aureus* pneumonia mouse models (Hua et al., 2014; Sause et al., 2016).

Furthermore, scFvs to the spirochete *Borrelia* have been found to have direct bactericidal effects (Larocca et al., 2008). Similarly, an anti-idiotype antibody that mimics yeast killer toxin has also been described to have direct cell-killing effects against an array of broad spectrum bacteria along with yeast, fungi and mycobacteria (Conti et al., 2000). Such antibodies may overcome the necessity for a competent host immune response for efficacy. More often, however, antibacterial antibodies rely on recruitment of varying antibody-directed immune effector functions such as; initiating antibody-directed cellular cytotoxicity (ADCC), activating the complement cascade, and inducing oxidative bursts. Moreover, all antibody variable regions are able to catalyze redox reactions resulting in the generation of potent oxidizing agents that directly harm bacteria (Wentworth et al., 2003). This process causes inflammation which further enhances the immune response by recruitment of immune system elements (Babior et al., 2003).

#### Antibody-Conjugates

Different antibody formats consisting of an antibody binding fragment conjugated to varying effector molecules like radionuclides (radioimmunotherapy/RITs), drugs (antibody-drug conjugates/ADCs), toxins (immunotoxins) and enzymes (antibody-directed enzyme prodrug therapy/ADEPT) are referred to as antibody conjugates. Conjugated mAbs are used to deliver one of these effector molecules specifically to infected cells while limiting the side effects of off-target drug delivery (Weiner et al., 2012).

##### Radioimmunotherapy/radioimmunoconjugates

The first effective form of targeted cancer therapy was^131^I, a β-particle emitter that also emits small amounts of γ-radiation. It was used for treatment of thyroid cancer because of its specificity for elemental iodine in the thyroid. The success of this treatment led researchers to explore various carrier molecules including mAbs which could be utilized for targeted delivery of radioisotopes to infected cells (Weiner, 2015). The first 2 FDA approved conjugated antibody therapies, were radioimmunotherapies, both for the treatment of hematological malignancies. These RITs were ^90^Y-ibritumomab tiuxetan and ^131^I-tositumomab, both targeting CD20 for treatment of relapsed and/or rituximab-refractory follicular or low-grade lymphomas. There are a number of other RITs in development, several of which target solid tumors (Weiner, 2015). Surface receptors (non-internalizing) are sufficient for treatment with RITs as internalization is not required by all RITs to induce killing of target cells (Illidge and Johnson, 2000). In addition, while most antibody-based therapies rely on targets with high binding affinity, RITs may be more effective against diseased cells with lower binding affinity targets; therapeutic proteins with high binding affinity will likely bind the first target receptor it contacts, predicted to be on the periphery of the tumor, and given its high affinity, will not penetrate deeper into the tumor thus resulting in radiation exposure to a higher number of healthy cells circulating around the tumor. However, lower binding affinity targets may permit better tumor penetration by allowing the therapeutic protein to move from one target expressing cell to the next, within the tumor microenvironment (Allen, 2011).

Whilst the use of mAbs has significantly improved specificity of radiotherapies many challenges persist limiting its widespread clinical application. Radioisotopes are in a continual state of decay harming healthy tissue during circulation or taken up non-specifically (Pouget et al., 2011). Bone marrow in particular is highly sensitive to radiation and is therefore affected by circulating RITs. In addition, the kidney and liver are exposed to high doses of radiation due to their roles in clearing RITs and free radioisotopes, resulting in a limited amount of radiation being delivered to target sites, even with the use of very specific targeting agents (Pouget et al., 2011). Further, the actual production of RITs is highly complex and requires experienced nuclear-medicine physicians due to the dangers of radiation poisoning. To overcome these challenges, many ongoing studies are assessing novel radioisotopes like α-emitters which deliver ionizing radiation adjacent, within up to a few cell diameters, to diseased cells consequently restricting radiation exposure to target cells and reducing exposure to neighboring benign cells (Allen, 2011). Current clinical and preclinical studies on such α-particles are ongoing and have the potential to significantly enhance RITs (Weiner, 2015).

There are presently no clinically approved RITs for treatment of infectious diseases, but studies have demonstrated promising progress in this field. The potential efficacy of RIT against *C. neoformans* (CN) has been of particular focus (Dadachova et al., 2003). Due to the current HIV pandemic, CN has emerged as a major fungal disease-causing life-threatening meningoencephalitis in 6–8% of AIDS patients. Further, CN infections are usually incurable in immunocompromised patients as current drug treatments are unable to eradicate the infection in this population (Casadevall et al., 1993; Currie and Casadevall, 1994). The α-particle emitter Bismuth-213 (^213^Bi) has been proposed for use in certain solid cancers and single-cell disorders and is currently in phase I/II clinical trials for treatment of leukemia patients (Boll et al., 1997; Sgouros et al., 1999; Adams et al., 2000; Mcdevitt et al., 2000). Consequently, a RIT comprising either of two radioisotopes, a high-energy β-emitter Rhenium-188 (^188^Re) or ^213^Bi and the capsule-specific mAb 18B7, were assessed with the CN mouse models. 18B7 has no inherent fungicidal activity by itself, but when conjugated to either ^188^Re or ^213^Bi, resulted in decreased fungal burden and prolonged survival in RIT treated mice (Dadachova et al., 2003).

The positive results of the CN study has prompted researchers to conducted a second investigation assessing the RIT approach in animal models of *Streptococcus pneumonia* (Dadachova et al., 2004). In this study the ^213^Bi radioisotope was conjugated to mAb D11, which has specific binding of pneumococcal capsular polysaccharide 8 (PPS8) and is protective against this serotype in multiple strains of mice and infection models (Zhong et al., 1999; Burns et al., 2003). ^213^Bi was specifically chosen over β-emitters for its short half-life that allows delivery of substantial amounts of radiation in a shorter time which is imperative when targeting rapidly dividing cells like pneumococci. Treatment of *S. pneumonia* with ^213^Bi-D11 RIT demonstrated bacterial killing in a dose-dependent manner and increased survival in *S. pneumonia-*infected C57BL/6 mice (Dadachova et al., 2004).

Furthermore, RIT has been reported to significantly decrease the number of HIV-infected cells *in vivo*, via conjugation of ^213^Bi and ^188^Re radioisotopes to gp41 (HIV-envelope protein)-specific mAb 246-D (Dadachova et al., 2006). A major advantage of using RIT as a treatment for viruses is that the RIT itself does not neutralize the virus, consequently reducing the probability of applying selection pressure that may result resistant strains forming (Dadachova et al., 2006). Recently, the potential therapeutic utility of RIT in combination with antiretroviral drugs (ARVs) has been demonstrated as a novel treatment for the eradication of HIV (Dadachova and Casadevall, 2014).

The morphological similarities observed between granuloma formation and that of a solid tumor, a highly fibrotic and hypoxic microenvironment, suggests this type of treatment may prove useful in TB patients (Datta et al., 2015). Furthermore, granulomas and solid tumors have functionally abnormal vasculature resulting in impaired small molecule distribution, exhibiting a wide range of variation in spatial drug distribution with majority of drugs accumulating in the periphery of the tumor/granuloma and not penetrating into the central diseased tissue. There are currently no RITs approved for the treatment of TB but given that granuloma penetration is a major challenge, RITs with a wider radiation field may be able to bind to the cells on the outside of the granuloma and still elicit killing of bacilli within the granuloma. Additionally, other forms of immunotherapy used for treatment of solid tumors may be applicable to TB granulomas; one important example is bevacizumab, an anti-VEGF antibody used to normalize tumor vasculature in solid tumors. A study conducted in rabbit TB models demonstrated the ability of bevacizumab to normalize the abnormal vasculature of granulomas, resulting in reduced hypoxia and increased small molecule drug penetration into the central granuloma (Datta et al., 2015).

##### Antibody-drug conjugates (ADCs)

Antibody-drug conjugates combine the specificity of mAbs with the cytotoxic potential of drugs (Weiner, 2015). Currently there are two FDA- approved chemotherapeutic ADCs, Ado-rastuzumab, a HER2^+^-targeting conjugate that exerts cytotoxicity via inhibition of microtubule assembly, and Brentuximab Vedotin, a CD30-targeting mAb conjugated to an antimitotic drug for the treatment of Hodgkin (NCT00848926) and anaplastic large cell lymphoma (NCT00866047) (Amiri-Kordestani et al., 2014; Herrera et al., 2018). This CD30-specific ADC, although approved for cancer treatment, has also been tested as an anti-viral ADC for the treatment of HIV (Hogan et al., 2018). CD30 is part of the TFN receptor superfamily, expressed on tumor cells and on a small population of lymphocytes in healthy individuals (Stein et al., 1985; Falini et al., 1995). Additionally, CD30 has been associated with HIV disease progression, with cell-associated HIV-1 RNA being considerably enriched in CD30 expressing CD4+ T cells. The significant reduction in HIV-1 DNA in PBMCs from HIV-infected donors by the ADC, suggests CD30 maybe a potential therapeutic target for HIV (Hogan et al., 2018).

Unlike RITs all ADC’s require internalizing targets to elicit their effector functions thus only targets which undergo receptor-mediated endocytosis may be used for ADC treatment (Trail et al., 2017). The distribution of receptors on diseased cells and tissue is also an important consideration when choosing a target for ADC’s. For diseases where penetration is not an issue, such as hematological cancers and other blood borne diseases, targeted therapeutics may easily bind to their target cells thus even a moderately expressed target may be sufficient for effective killing. However, for solid tumors, TB granulomas and other difficult to penetrate diseases it is important that the therapeutic protein have an increased serum half-life to allow sufficient time for uptake into diseased tissue (Allen, 2002). Furthermore, the frequency of target receptor internalization/recycling may also affect the binding of the therapeutic protein. For targets with fast turnover rates, changes in surface receptor expression have little impact on the accumulation of the ADC in target cells whereas targets with slow turnover rates show reduced efficacy with decrease surface receptor expression (Sadekar et al., 2015).

Recently, an antibody-antibiotic conjugate (AAC), Thiomab combining essential characteristics of a mAB and an antibiotic has been shown to be an effective therapy against *S. aureus* (Lehar et al., 2015). This ACC works similarly to ADC’s in that it uses and antibody to deliver an antibiotic payload to bacteria (Mariathasan and Tan, 2017). As with ADC’s the antibody, linker and payload used in the design of the ACC play critical roles in the binding, stability, and effector functions, respectively. A mAb, directed against β-*N-*acetylglucosamine cell-wall teichoic acid (β-GlcNAc) residues on the cell wall of *S. aureus* was chosen as this target is highly expressed on *S. aureus* but not on mammalian cells. For this ACC, a dimethyl DNA31, referred to as dmDNA31, was the chosen antibiotic as it possesses a functional group amenable to conjugation, it is active against *S. aureus* in both its dormant and active state and has potent bactericidal activity. Finally, the linker selected for the ACC was a MC-ValCit-PABQ linker consisting of caproic acid and maleimide for attachment to the antibody, a protease-cleavable dipeptide, valine citrulline and a novel salt, PABQ for attachment of the antibiotic. Thiomab effector function comprises killing of intracellular bacteria via intracellular release of dmDNA31 and internalization of extracellular bacteria through rapid opsonization. Given the rapid emergence of antibiotic resistance and lack of discovery of new antibiotics the ACC avenue provides opportunity for developing novel therapeutics to treat infectious diseases (Mariathasan and Tan, 2017).

##### Immunotoxins

A classical IT is comprised of a binding domain and a toxic protein domain originally attached by chemical conjugation and later recombinantly fused. The binding domain of an IT may be natural ligands (growth factors, surface receptors), an antibody, or a recombinant derivative (Barth, 2009). To be effective, an IT needs to bind to a surface receptor of an infected cell, undergo endocytosis and intracellular trafficking of its catalytically active subunit to the cytosol where it induces cell death. Additionally, the natural binding domains of the toxins need to be removed so that cellular uptake may be controlled by the specificity of the mAb alone. Historically, bacterial- or plant-derived toxins have first been used to generate recombinant ITs (rITs). Their recognition as foreign antigens by the human immune system, has resulted in induction of neutralizing antibody responses in patients. To reduce immunogenicity, (a) B and T cell epitopes have been removed from the protein toxins, and (b) the bacterial/plant toxins have been replaced by human apoptosis-inducing enzymes which are non-toxic until delivered intracellularly to the target cell (Jordaan et al., 2018). As such, targeted human cytolytic fusion proteins represent the 4th and most recent generation of anticancer immunotherapeutics, with a plethora of candidates such as granzyme B, angiogenin, DAP-K and Map-tau currently under preclinical investigation (Hristodorov et al., 2013; Amoury et al., 2016; Lilienthal et al., 2016; Akinrinmade et al., 2017).

Majority of the focus on developing ITs has been for treatment of cancer, however, there are also those that target viruses (Spiess et al., 2016). Anti-viral ITs interfere with the infection cycle of viruses by targeting virus entry, intracellular replication, viral particle formation and cellular extrusion or by modulating the anti-viral cellular immune responses (Zhu et al., 2015). In addition, anti-viral ITs have a capacity to kill viral infected cells (Spiess et al., 2015; Zhu et al., 2015) and because target molecules are often encoded by the virus itself, off-target killing of cells expressing low levels of the target receptor is greatly reduced compared to anti-cancer immunotoxins. Furthermore, interference of protein synthesis in infected cells prevents viral dissemination and destroys reservoirs of latently infected cells (Geoghegan et al., 2015) which pertains to diseases such as HIV, where current therapies only target active viral infections and the lack of eradication of latently infected cells results in patients needing lifelong therapy (Kennedy et al., 2006). ITs comprising anti-HIV capsid protein gp120 mAb linked to a truncated form of *Pseudomonas exotoxin A* (PE) display specific and potent cytotoxicity against HIV replication in monocyte-derived macrophages and PBMC’s (Ashorn et al., 1991; Berger and Pastan, 2010). Mice studies utilizing gp120-specific ITs in combination with standard therapy showed significant improvement over standard therapy alone but was unable to eradicate the disease (Goldstein et al., 2000), probably as a result of immunogenicity-related neutralization. A major complication of immunotoxin-based treatment of HIV is the limited expression of HIV-proteins in latently infected cells, engendering this cell population invisible to the immunotoxin. Combination therapy of ADC’s with drugs such as latency-revering agents, may increase the target receptor expression on latently infected cells enhancing susceptibility to ADC-induced toxicity (Perreau et al., 2017). Furthermore, two PE-based ITs YC15-PE38 and 2014-PE38, have been developed for the specific killing of Kaposi’s sarcoma-associated herpesvirus (KSHV)-infected cells and target KSHV lytic glycoproteins. Both ITs impede the construction of infectious KSHV particles and specifically kill KSHV-infected cells (Cai and Berger, 2011; Chatterjee et al., 2012).

In recent times, antibodies isolated from members of the camelid family have been used to develop ITs directed against herpes-simplex virus 2 (HSV-2) (Geoghegan et al., 2015). These antibodies are termed VHH as they consist of the variable domains of antibody heavy-chains and lack light chain and CH1 domains (Hamers-Casterman et al., 1993). VHH share a large degree of homology with mammalian variable domains and have enhanced tissue penetration due their smaller size (Cortez-Retamozo et al., 2002). A VHH, R33 antibody with specific binding to viral cell surface glycoprotein D on HSV-2 was identified using phage display. R33 alone did not have any neutralizing effect on HSV-2, however, in combination with the cytotoxic domain of PE, this IT demonstrated extremely efficient killing of HSV-2-infected cells *in vitro*.

In addition to anti-viral ITs, the targeted delivery of granzyme B (Araki et al., 2013) to *Plasmodium falciparum* provided the first evidence of granzyme B’s anti-parasitic effects on malaria (Kapelski et al., 2015). The merozoite surface protein 4 (MSP4)-targeting IT induced enhanced inhibition of parasite growth in infected erythrocytes and although the exact mechanism of action is unknown the evidence strongly suggests that malaria-specific ITs are valuable drug candidates for treatment of multidrug resistant *P. falciparum* strains.

Antibody drug conjugates has significantly improved in efficacy over the last few years, however, its therapeutic application is still limited and require further improvements. All ADC’s currently approved for therapy target tumor associated antigens not tumor specific antigens thus resulting in undesired toxicity that limits its therapeutic application. New generation ADC’s should focus on disease-specific targets and will most likely require payload that do not interact with cells unless conjugated to an antibody. The major challengers in developing ADC’s are identifying appropriate targets, designing chemically stable linkers and choosing an appropriately cytotoxic drug (Lin et al., 2018). All of these factors require extensive optimization to fully improve the efficacy and tolerability of ADC’s thus understanding each of these components is key to developing new generation ADCs.

## Conclusion

In spite of the conventional association of the term “immunotherapy” with the treatment of cancer, indeed publication records are indicative of this bias, this approach is fast-establishing itself within the armory of therapeutics applicable across the disease spectrum. Herewith, the versatility of the strategies that distinguish the immunotherapeutic approach have been described, with an emphasis on therapies that enhance T cell effector function as well as ligand-based therapies that neutralize or eliminate diseased cells. Although not within the scope of this review, natural killer (NK) cell immunotherapies, particularly CAR-modified NK cells and bispecific killer cell engagers (BiKEs), are rapidly gaining attention for application in cancer and HIV (Gleason et al., 2014; Rezvani and Rouce, 2015; Schmohl et al., 2016; Liu et al., 2017). Although the development of vaccines, whether therapeutic or prophylactic, still remains a major focus for infectious diseases such as HIV, based on the number and progress of preclinical studies, the development of immune checkpoint inhibitors and antibody-based therapies are emerging as promising approaches. T cell engineering has transformed adoptive therapies; CAR T cells in particular have gained massive recognition in the cancer field and although not initially successful in clinical trials, second-generation anti-HIV CAR T cells are being explored for their potential to provide long-term protection (Zhen et al., 2017; Wagner, 2018). In addition, it is evident that advances in antibody-conjugates against cancer have paved the way for similar strategies in infectious diseases, especially HIV and various bacterial and fungal infections. In this regard, an onslaught of novel agents and a significant increase in pre-clinical studies in various disease models can be expected.

Despite these encouraging results, ultimately, the high cost of these agents will necessitate comprehensive evaluations of the economic sustainability on healthcare systems, particularly in low- and middle-income countries where the burden of diseases such as HIV and TB remain significant. In addition, the common adverse effects amongst T cell-based therapies, specifically neurotoxicity and CRS, although manageable, remains a major challenge during patient care. Importantly, not all patients respond to immunotherapy, and in cancer, this is attributed to various resistance mechanisms. Combination immunotherapy may provide a solution; a multipronged approach to a curative treatment that has been heralded as the next wave of therapeutic strategies for cancer, proving successful with the recent case of complete durable regression of metastatic breast cancer (Morrissey et al., 2016; Zacharakis et al., 2018). Combinatorial strategies that promote an immunogenic tumor microenvironment by introducing existing chemotherapeutic agents with immune checkpoint inhibitors are fast gaining clinical recognition (Sharma and Allison, 2015). Recently, a mathematical model was developed to predict the efficacy of the combination of a cancer vaccine with an immune checkpoint inhibitor (Lai and Friedman, 2017). The establishment of such tools will be essential to provide rational insight to the advancement of combination immunotherapy. Clinical studies evaluating the configuration of these combinatorial regimens will be equally important (Schmid et al., 2018). Certainly, a combinatorial approach is central to achieving an HIV cure (Perreau et al., 2017). Of note, despite the incredibly diverse application of mAbs for infectious diseases, its relative underutilization in comparison to cancer may be attributable to the high cost compared with small molecule drugs or vaccines. Consequently, it is possible that immunotherapies may be relegated to adjunct or interim intervention strategies.

The age of immunotherapies has revolutionized cancer treatment. However, there is still much room left for improvement and efforts are ongoing to address better patient outcomes (Mohindra, 2018). With the growing understanding of the biological etiology of diseases, the current portfolio of immunotherapies is expected to expand significantly and may be, just as groundbreaking in combating infectious diseases.

## Author Contributions

KN and TN contributed equally to the development and writing of the paper, reviewing relevant literature, and preparation of figures in the paper. SC contributed to the writing of the paper and provided the table on vaccines. SB provided substantial, direct, and intellectual contribution to the work. All authors consented for publication.

## Conflict of Interest Statement

The authors declare that the research was conducted in the absence of any commercial or financial relationships that could be construed as a potential conflict of interest.

## Footnotes

1 www.clinicaltrials.gov
